# Targeting Gliomas with Beta-Amyloid-Specific Dyes: A Novel Approach for In Vivo Staining and Potential Therapeutic Applications

**DOI:** 10.3390/ijms262110450

**Published:** 2025-10-28

**Authors:** Lilia Kucheryavykh, Jescelica Ortiz Rivera, Boris Ermolinsky, Vassiliy Tsytsarev, Lynnette Cary, Janaina Alves, Adriana Reyes, Noelis de la Cruz-Rivera, Kevin Rosa Gonzalez, Felix Narvaez Irizarry, Tyrel R. Porter, Mikhail Inyushin

**Affiliations:** 1 Department of Biochemistry, Universidad Central del Caribe (UCC), Bayamón, PR 00960, USA; lilia.kucheryavykh@uccaribe.edu (L.K.); 416jortiz@uccaribe.edu (J.O.R.); janaina.alves@uccaribe.edu (J.A.); 423krosa@uccaribe.edu (K.R.G.); 423fnarvaez@uccaribe.edu (F.N.I.); 122tporter@uccaribe.edu (T.R.P.); 2 Department of Biology, University of Texas Rio Grande Valley (UTRGV), Brownsville, TX 78539, USA; boris.ermolinsky@utrgv.edu; 3 Department of Pharmacology and Molecular Therapeutic, Uniformed Services University of the Health Sciences (USUHS), Bethesda, MD 20814, USA; vassiliy.tsytsarev.ctr@usuhs.edu (V.T.); lynnette.cary@usuhs.edu (L.C.); 4 Department of Biology, Universidad Interamericana de Puerto Rico en Bayamón (INTER Bayamón), Bayamón, PR 00957, USA; adrv16@gmail.com (A.R.); ndelacruz0350@interbayamon.edu (N.d.l.C.-R.)

**Keywords:** glioma, beta-amyloid, in vivo staining, boron-containing dyes, boron neutron capture therapy (BNCT)

## Abstract

Gliomas, the most common primary brain tumors, present significant diagnostic and treatment challenges due to their infiltrative nature and heterogeneity. Our previous research revealed that glioma tumors in both animals and humans accumulate beta-amyloid protein (Aβ), detectable through immunohistochemical methods or staining with amyloid-specific dyes. We hypothesize that beta-amyloid-specific dyes could serve as glioma markers, potentially enabling the delineation of glioma tumors or targeted therapeutics delivery. In this study, the specificity and blood-brain barrier permeability of two fluorescent beta-amyloid-specific dyes, Brilliant Blue G (BBG) and BODIPY-based Amyloid Probe-1 (BAP-1), were evaluated in C57Bl/6 mouse glioma implantation models using GL261 and KR158 glioma cells. The findings demonstrate that both BBG and BAP-1 selectively stain gliomas, providing a clear contrast from normal brain tissue. The study results open avenues for further development of glioma visualization methods and targeted therapeutic delivery strategies for clinical applications.

## 1. Introduction

Glioblastoma (GBM), classified as a CNS WHO grade 4 glioma, is the most prevalent and aggressive form of primary malignant brain tumor. Despite significant research efforts, there remains no effective cure, and GBM is associated with a short median survival time, despite surgery [1].

Recently, we have reported that GBM tumors in both mice and humans contain beta-amyloid protein (Aβ). This protein can be detected using immunocytochemistry and traditional amyloid-binding dyes, such as Congo Red and Thioflavin [2,3]. Beta-amyloid (Aβ) aggregates are characterized by highly ordered cross-β sheet structures, which form insoluble fibrils and plaques in the brain in Alzheimer’s disease. Previous in vitro experiments demonstrated that glioma cells secrete small amounts of Aβ peptides, which are recognized by antibodies targeting the peptide’s terminal regions [4].

However, it has also been suggested that Aβ found in glioma tumors may originate from external sources, particularly through the bloodstream [2]. This hypothesis is supported by experiments involving photothrombosis using Rose Bengal, which induced localized ischemia in the brain cortex. These studies showed initial Aβ accumulation in thrombosed blood vessels, with subsequent diffusion into surrounding brain tissue [2]. Furthermore, in Aβ-secreting cells, amyloid precursor protein (APP), its metabolites, and key enzymes responsible for APP cleavage are predominantly associated with extracellular vesicles [5]. In contrast, within glioma tumors, Aβ peptides are primarily located in membrane fractions rather than freely dispersed in the cytoplasm or extracellular space [2], suggesting that glioma cells might not synthesize Aβ themselves, but instead absorb it from the tumor microenvironment. In terms of total brain Aβ content, normal (control) human brains contain, on average, about 1.7 mg of amyloid-beta in the cortical grey matter, compared to much higher amounts (~6.5 mg) in AD-affected brains [6]. This reflects that healthy brains maintain relatively low amounts of amyloid deposits and predominantly soluble forms of Aβ. ELISA measurements in a mouse model demonstrated about a 142% increase in Aβ40 levels in glioma tumor tissue relative to healthy brain tissue from the same animal [2]. In biochemical terms, this translates into more than double the concentration of Aβ40 peptide in glioma tissue when normalized to protein content.

Additionally, many other glioma-associated proteins, for example, glioma-associated extracellular matrix (ECM) proteins and beta-amyloid aggregates, share a key structural feature: the presence of β-sheet-rich assemblies. ECM proteins in glioma, such as collagens, fibronectin, laminins, and tenascin-C, can also adopt β-sheet-rich conformations or form fibrillar assemblies with organized β-sheet content, contributing to the fibrous scaffold of the tumor microenvironment [7,8,9]. For example, type I collagen—prominent in glioma ECM—forms rigid fibrils stabilized by β-sheet interactions, which influence both mechanical properties and cell adhesion patterns critical for glioma invasion. The current data suggest that gliomas may present aggregate-prone microenvironments analogous to those found in neurodegenerative conditions, additionally the use of amyloid-sensitive statins.

Despite accumulating evidence of Aβ presence in GBM, the biological significance of this phenomenon remains poorly understood [10]. Nonetheless, we propose that amyloid accumulation in GBM may serve as a potential marker for tumor identification and treatment. This study analyzes the potential of amyloid-specific dyes for future clinical applications, specifically focusing on (1) intraoperative visualization of glioblastoma tumors and (2) targeted destruction of tumor cells.

### 1.1. Intraoperative Visualization

If an amyloid-binding dye is safe, able to cross the blood-brain barrier (BBB), and fluorescent, it could be used intraoperatively to delineate tumor margins with a simple intravenous injection. Fluorescent enhancement can aid surgeons in identifying tumor borders, thus increasing the likelihood of achieving gross total resection. Currently, 5-aminolevulinic acid (5-ALA) and fluorescein sodium (SF) are two widely used dyes for this purpose [11,12,13].

5-ALA is a natural amino acid that is metabolized into the fluorescent compound protoporphyrin IX (PpIX), which accumulates preferentially in tumor cells. Under red-violet light (640–710 nm), PpIX fluoresces, enabling more accurate resection. Clinical use of 5-ALA has increased the gross total resection rate in gliomas from 36% (white-light surgery) to 65% [13,14].

In contrast, SF is a non-targeted tracer that typically does not cross the intact BBB. However, in high-grade gliomas (HGGs), the BBB is disrupted, allowing SF to accumulate in tumor tissue and emit yellow-green fluorescence at around 560 nm. SF is inexpensive, non-toxic, and increases GTR rates to 55–75% in contrast-enhancing tumors [13,14]. Dual use of both 5-ALA and SF can be used to guide the initial phase of resection, while 5-ALA was used to visualize tumor spots within the surgical cavity, to achieve gross total resection [15], while it was shown that 4-ALA may discriminate between molecular subtypes of glioma [16]. ICG (indocyanine green), another dye, is mainly used for vascular visualization and has potential toxicity concerns [11,17,18].

While effective, these dyes have limitations. 5-ALA is costly, phototoxic, and requires dark operating room conditions due to its low fluorescence yield. SF, although safer and cheaper, lacks specificity for glioma cells. Therefore, there is a need for new fluorescent markers that are non-toxic, cost-effective, capable of crossing the BBB, and highly specific to glioma tissue.

### 1.2. Targeted Tumor Destruction via Boron-Neutron Capture Therapy (BNCT)

Beyond visualization, amyloid-specific dyes may offer therapeutic benefits. Specifically, if a dye incorporates the non-radioactive isotope boron-10 (B10, which is about 20% of all boron isotopes and is non-radioactive), it can be used in BNCT. This approach involves administering B10-containing compounds that preferentially accumulate in tumor cells. When exposed to a beam of low-energy (thermal) neutrons, B10 undergoes nuclear fission, releasing high-energy alpha particles and lithium-7 nuclei. These particles selectively destroy nearby tumor cells while sparing healthy tissue.

Successful BNCT requires:

1. High B10 concentration within tumor cells;
2. Tumor-to-normal tissue and tumor-to-blood concentration ratios exceeding 3–5;
3. Minimal toxicity of boron carriers [19].

Since the 1950s, BNCT has progressed from using simple boron compounds like boric acid and borax [20,21,22] to more targeted agents, such as L-p-boronophenylalanine (L-10BPA) and sodium borocaptate (BSH). However, L-10BPA has low boron content, necessitating repeated dosing, while BSH lacks tumor specificity and may cause collateral damage. Recent advances in hospital-based neutron sources have enabled broader clinical testing of BNCT in the treatment of various cancers [20,23,24].

**Figure 1:**
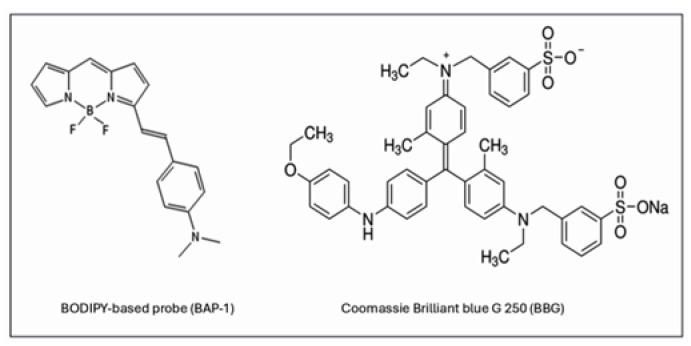
**BAP-1 and BBG chemical structure**.

In light of these developments, we investigate the use of Aβ-specific dyes as potential dual-function agents for GBM, serving as both tumor markers and boron carriers for BNCT. In this study, we explore the in vivo staining of gliomas using two Aβ-binding dyes: Brilliant Blue G (BBG, Coomassie Brilliant Blue, not containing B10) and BODIPY-based Amyloid Probe-1 (BAP-1, containing B10) (Figure 1). By analyzing their fluorescence distribution in tumors and surrounding tissues, we aim to explore the potential of these dyes for future development as tools in fluorescence-guided surgery and as boron carriers in BNCT.

## 2. Results

### 2.1. BBG Selectively Accumulates in Glioma Tissue

Intraperitoneal (IP) injection of BBG in animals with induced glioma tumors resulted in visible reddish-brown coloration of the tumor tissue within 30 min post-injection (Figure S1), suggesting that BBG binds to specific tumor-associated proteins [25,26]. BBG is known to be inexpensive, non-toxic, capable of crossing the blood-brain barrier (BBB) shortly after administration, and exhibits strong fluorescent properties [25]. The tumor mass showed clear demarcation, visible even without the use of microscopy.

**Figure 2:**
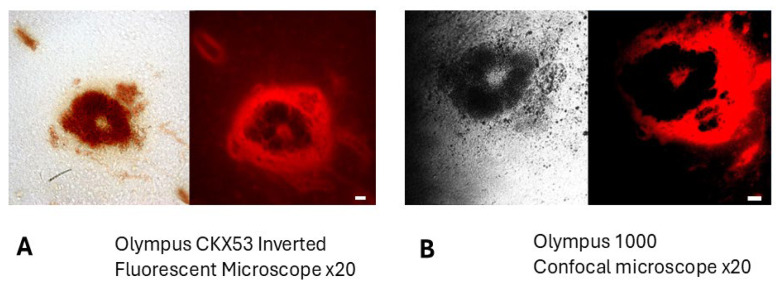
**Fluorescence Detection of BBG in Brain Tumors Using Fluorescent and Confocal Microscopy.** Intraperitoneal injection of BBG enables visualization of brain tumors through fluorescence, detectable with both widefield fluorescence (**A**) and confocal microscopy (**B**). These micrographs show brain sections from C57Bl/6-GL261 mice with implanted gliomas after IP injection of BBG. Thirty minutes after injection of 100 µL of BBG (10 mg/mL in PBS), the brains were harvested. Following fixation in 4% PFA and dehydration in sucrose gradients, 70 µm frozen sections were prepared. Brightfield and fluorescent images were acquired using TRITC HYQ filter sets (Excitation: 530–560 nm, Emission: 590–650 nm). Scale bar: 100 µm.

Microscopic analysis of brain slices containing tumors further revealed well-defined tumor margins and localized tumor foci in the brain parenchyma, appearing as brown-colored regions under brightfield microscopy. (Supplemental Figure S1) Fluorescence imaging (Figure 2A) and confocal microscopy (Figure 2B) of the same tissue slices revealed significant accumulation of fluorescence at the tumor periphery and near the tumor margins, suggesting the presence of filamentous proteins or peptides concentrated in these regions. Confocal microscopy, which reduces out-of-focus blur inherent in conventional fluorescence microscopy, confirmed that the observed fluorescence pattern was not an imaging artifact.

While BBG is capable of staining individual tumor cells, it appears that after IP injection, it preferentially binds to filamentous proteins or peptides concentrated in the peripheral and peritumoral regions. This binding behavior is consistent with previous reports of BBG interaction with beta-amyloid filaments and its role in promoting Aβ oligomer aggregation [27], similar to the triarylmethane dye crystal violet, which primarily binds short beta-amyloid filaments [28].

**Figure 3:**
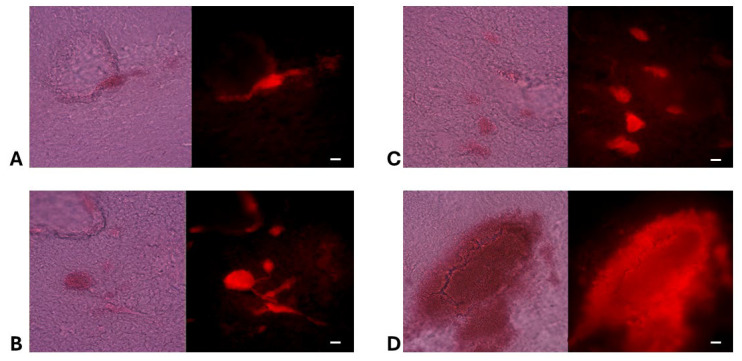
**Hematoxylin–Eosin and BBG Fluorescence in Glioma-Bearing Brain Slices**. Frozen brain sections (70 µm) containing C57Bl/6-GL261 glioma implants were prepared from animals sacrificed 30 min after intraperitoneal injection of 100 µL BBG (10 mg/mL in PBS). Following fixation in 4% paraformaldehyde and sucrose dehydration, sections were stained with H&E. Fluorescence from BBG—shown in red—was imaged on an Olympus CKX53 using TRITC HYQ filters (Excitation: 530–560 nm; Emission: 590–650 nm). Representative images illustrate small tumor formations at the invasion margin (**A**–**C**) and the primary implanted tumor (**D**). Brightfield H&E images appear in the left panel of each figure. Scale bar: 200 µm.

Following hematoxylin-eosin (H&E) staining, the fluorescence signal was retained only within the glioma cells of the tumor, while the peripheral fluorescence disappeared, as confirmed by confocal microscopy (Figure 3, see also high-resolution Supplement figures).

**Figure 4:**
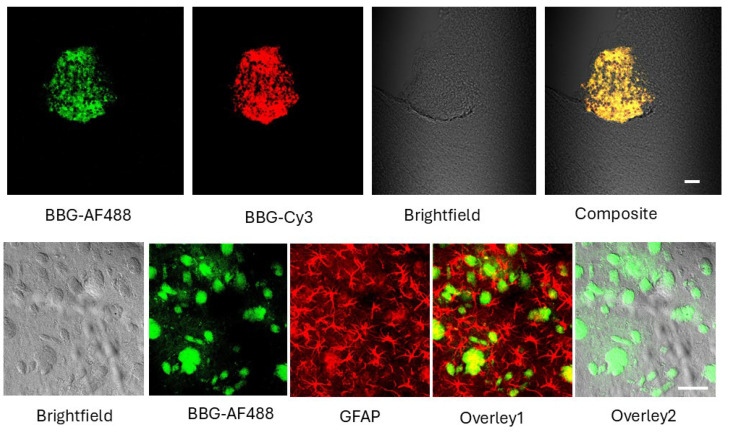
**BBG staining of fixed glioma brain sections.** Upper panel: Representative images of fixed brain sections from C57Bl/6 mice bearing GL261 gliomas stained with 0.1 mM BBG in PBS and rinsed with ethanol. Brightfield and fluorescence images acquired with Alexa Fluor 488 and Cy3 filter sets illustrate the broad excitation/emission properties of BBG. BBG produced robust labeling of compact clusters of cells with morphology consistent with tumor tissue. Lower panel: Double labeling of glioma sections with BBG and GFAP. Fixed glioma-bearing sections were first immunostained with a rabbit monoclonal anti-GFAP antibody directly conjugated to Alexa Fluor^®^ 594 (detected in the Texas Red channel), followed by post-staining with 0.1 mM BBG. Brightfield and fluorescence images were acquired using Alexa Fluor 488 (BBG) and Texas Red (GFAP) channels. Overlays demonstrate that BBG staining in fixed slices co-localizes with GFAP-positive glioma cells while also lightly labeling additional material within the tumor. Scale bar: 100 µm.

Besides staining glioma tumors after IP injection, BBG can stain tumors by directly applying this stain to fixed slices. We incubated fixed glioma-bearing brain slices with BBG (0.1 mM, 30 min). Since these slices are fixed, BBB penetration cannot occur. Nonetheless, BBG produced robust labeling of compact clusters of cells with morphology consistent with tumor cells (Figure 4 upper panel). This finding indicates that BBG binds glioma-associated structures directly, not only through passive vascular leakage. Because of BBG’s broad excitation/emission spectrum, we verified its fluorescence signal in two independent channels (Alexa Fluor 488 and Cy3 filter sets). Reproducible detection across both channels confirmed that the observed signal reflects true BBG fluorescence (Figure 3 upper panel) rather than autofluorescence.

We also performed double-labeling with GFAP, a well-established marker of astrocytomas and glioblastomas in rodent models (Figure 4, lower panel). BBG co-localized with GFAP-positive glioma cells, supporting the conclusion that BBG identifies tumor cells. Although BBG also lightly stained some extracellular or stromal material within the glioma (possibly tumor matrix), its predominant localization was within GFAP-positive tumor cells.

### 2.2. BAP-1 Preferentially Accumulates in Glioma Cells with High Contrast

**Figure 5:**
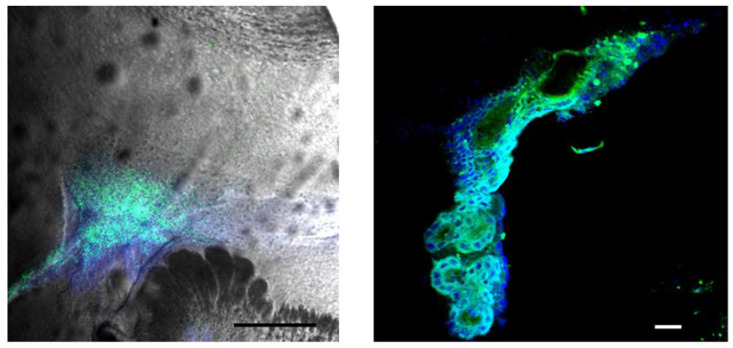
**Confocal Imaging of BAP-1–Stained Glioma in Mouse Brain Sections.** Intraperitoneal injection of BAP-1 enables selective staining of glioma tumors in the C57Bl/6-GL261 mouse model, visualized as red fluorescence. Confocal micrographs show 70 µm frozen brain sections prepared 30 min after IP injection of 100 µL BAP-1 (2 mg/mL in PBS). Brains were fixed in 4% paraformaldehyde, dehydrated through sucrose gradients, and sectioned, then counterstained with DAPI. Images were acquired using TRITC HYQ filters (Excitation: 530–560 nm; Emission: 590–650 nm). Tumor cells are presented in green arbitrary color, and nuclei are labeled with DAPI (blue). Scale bar: 1 mm (**left panel**) and 10 µm (**right panel**) (see also Supplement figure with separated DAPI and BAP-1 channels).

Thirty minutes after intraperitoneal injection, BAP-1 selectively accumulated in glioma cells, producing a high fluorescence contrast ratio (2404 ± 398, *n* = 10; mean ± standard error) compared to the surrounding tissue which exhibited no detectable fluorescence in paraformaldehyde -fixed brain sections (Figure 5). This indicates a high concentration of BAP-1 within glioma cells relative to adjacent normal brain tissue. The stained glioma cells emitted fluorescence near 650 nm, consistent with previously reported BAP-1 labeling of cerebral beta-amyloid plaques [28].

**Figure 6:**
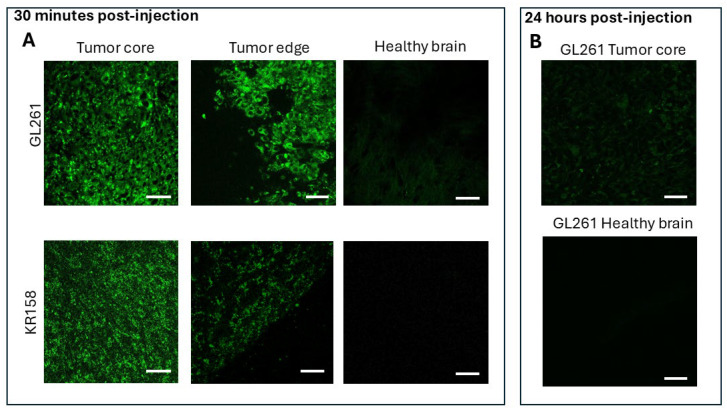
Confocal Imaging of BAP-1–Stained Glioma in Live Mouse Brain Sections. Intraperitoneal injection of BAP-1 enables selective staining of glioma tumors in the C57Bl/6-GL261 and C57Bl/6-KR158 mouse models, visualized as green fluorescence. Confocal micrographs show 300 µm live brain sections prepared 30 min after IP injection of 100 µL BAP-1 (2 mg/mL in 2% polyethylene glycol (PEG-300) in PBS). Images were acquired using TRITC HYQ filters (Excitation: 530–560 nm; Emission: 590–650 nm). Tumor cells are presented in green (pseudocoloring). Scale bar: 10 µm (see also Supplementary Table S1).

Confocal imaging of live brain slices further confirmed BAP_1 accumulation within tumor cells, enabling clear visualization of individual cells in both the tumor core and the invasion margin in GL261 and KR158 glioma models implanted in the cortex of C57Bl/6 mice (Figure 6). Only minimal fluorescence was observed in the surrounding healthy brain parenchyma. BAP-1 fluorescence highlighted tumor cells in both glioma models, with more intense accumulation in GL261 tumors compared to KR158. The intensity of fluorescence varied from cell to cell within the tumors, reflecting tumor cell heterogeneity in their ability to accumulate BAP-1. At 24 h post-injection, only trace amounts of fluorescence remained in the tumors, with no detectable signal in the healthy brain tissue, suggesting rapid clearance of BAP-1 or signal fading over time.

To optimize the dosing, 0.04 mg and 0.02 mg BAP-1 were analyzed, and tumor fluorescence was evaluated 30 min later in live brain slices. The 0.04 mg dose provided the highest glioma-to-background contrast (Supplementary Figure S2). Additional histological analysis revealed residual BAP-1 accumulation near the IP injection site (Supplementary Figure S3), which may pose a risk during subsequent neutron irradiation.

Tail-vein injections resulted in weak glioma staining (Supplementary Figure S3), likely due to first-pass hepatic metabolism reducing the tracer’s availability in systemic circulation [29].

## 3. Discussion

In this study, we confirmed the presence of Aβ in GBM and demonstrated the potential of two intraperitoneally administered fluorescent dyes—BBG and BAP-1—as potential clinical tools for glioma visualization and for boron delivery for BNCT. Our study was limited to high-grade glioma models (GL261, KR158). We acknowledge the importance of grade-specific Aβ accumulation and hope that our future studies will include gliomas of different grades.

Thirty minutes after intraperitoneal injection, both BBG and BAP-1 produced rapid, high-contrast labeling of glioma cells in the C57Bl/6-GL261 and -KR158 mouse models (Figure 2, Figure 3, Figure 4, Figure 5 and Figure 6). BBG provided the added advantage of visible brown staining under brightfield illumination, suggesting its potential for low-magnification or even naked-eye tumor demarcation during surgery. Further preclinical studies across multiple animal models, as well as dose optimization, are required to clarify its effects and validate its utility.

Confocal and widefield fluorescence imaging confirmed that BBG accumulation was more prominent at the tumor margin compared to the tumor core (Figure 2). Subsequent H&E staining, which involves placing tissue samples through a series of alcohols that dissolve extracellular aggregates of oligimeric Aβ, which are predominantly accumulated at tumor margins and in the peritumoral area, eliminated the peripheral fluorescence, leaving a weaker glioma-cell–associated signal, detectable only at higher exposure settings (Figure 3). Because of BBG’s broad excitation/emission spectrum it is even better detectable in high-resolution black-and-white Supplement figures of these tumors. We interpret this shift as evidence that BBG first binds to soluble oligomeric Aβ or other filamentous peptides in the peritumoral space—material that is subsequently washed away during the H&E staining process—before concentrating within tumor cells. Given that extracellular Aβ oligomers form soluble filamentous plaques in rodents [30], our data may represent the first detection of such soluble Aβ oligomers in the glioma microenvironment, as previously hypothesized [31,32]. In addition to in vivo administration, we also examined whether BBG can directly stain glioma tissue in the absence of vascular delivery. For this purpose, fixed glioma-bearing brain slices were incubated with BBG (0.1 mM, 30 min). Because these slices were fixed, blood–brain barrier penetration could not contribute to dye distribution. Nevertheless, BBG produced robust labeling of compact clusters of cells with morphology characteristic of tumor tissue (Figure 4, upper panel). This demonstrates that BBG is capable of binding glioma-associated structures directly, and that its tumor enrichment is not explained solely by passive vascular leakage. Owing to the broad excitation and emission spectrum of BBG, fluorescence was verified using two independent filter sets (Alexa Fluor 488 and Cy3). Consistent signal across both channels confirmed that the labeling represents true BBG fluorescence rather than autofluorescence. Furthermore, double-labeling experiments with GFAP, a well-established astrocytic and glioma marker in rodent models, revealed substantial co-localization of BBG with GFAP-positive tumor cells (Figure 3, lower panel). Although light staining of extracellular or stromal material within the tumor mass was also observed—potentially reflecting binding to matrix components—the predominant localization of BBG was within GFAP-positive glioma cells. This approach rules out BBB disruption as a confounding factor and supports the hypothesis that BBG interacts with glioma-specific cellular or extracellular structures.

There are also limitations: Although GFAP is a robust glioma marker, it does not label all glioma subpopulations (e.g., GFAP-negative tumor cells), and future work could incorporate additional markers (e.g., Sox2, Olig2). Also, a faint extracellular staining was found in the glioma tumor zone that requires further characterization to determine whether it reflects binding to amyloid-like deposits, extracellular matrix, or necrotic material.

Chemically, BBG is a triarylmethane dye whose color and spectral properties vary with pH: it appears red (λₐₓ 465) nm below pH 0, green (λₐₓ 620 nm) at pH 1, and bright blue (λₐₓ 595 nm) above pH 2. BBG binds noncovalently to protein amino and carboxyl groups, forming stable complexes even under acidic conditions [27]. When bound to filamentous proteins (e.g., keratin), its absorbance shifts from 465 nm to 595 nm—a property exploited in the Bradford assay to quantify protein concentration via increased absorption at 595 nm [33]. Although BBG’s absorption spectrum has been well characterized, its red fluorescence upon protein binding has been less documented. Similarly to crystal violet, which fluoresces when bound to proteins [34]—BBG exhibits a broad red-emission band when complexed (excitable across 330–380 nm or 450–490 nm, with emission maxima outside these ranges) [35,36,37,38]. In the current studies, the TRITC HYQ filters (Ex. 530–560 nm; Em. 590–650 nm), aligning with BBG’s red-fluorescent peak, were used [35,37].

The combination of brown coloration in the tumor bulk without visible staining of the peritumoral area, along with predominant fluorescence accumulation in the peritumoral region and lower fluorescence levels in the tumor core, likely reflects differences in pH between these regions and BBG’s pH sensitivity (Figure S1). BBG exhibits pH-dependent color changes due to its structural properties: it appears blue at pH ≥ 1.1 in ethanolic-phosphoric acid solutions or when bound to proteins, but shifts to reddish-brown in acidic (pH < 1.0) protein-free solutions [33]. The brown coloration of the tumor core with low fluorescence may therefore result from acidification of the tumor core and accumulation of unbound dye.

BBG’s affinity for filamentous Aβ has been well characterized. It functions as an Aβ-oligomer aggregation modulator, reducing Aβ-associated cytotoxicity in a dose-dependent manner by promoting the formation of off-pathway, non-toxic aggregates [33]. BBG specifically targets beta-amyloid protein in its filamentous form, with minimal effect on other amyloids. For instance, it affects diabetic amylin polymerization only at high concentrations [33]. Clinically approved as Coomassie Brilliant Blue for veterinary surgery since 2019, BBG is non-toxic (oral LD_50_ in rats > 5000 mg/kg) and crosses the blood–brain barrier efficiently [39].

BBG’s pharmacological and biochemical properties further support its clinical potential. In Alzheimer’s disease models, BBG improves cognition, inhibits Aβ-induced loss of filopodia and dendritic spines in hippocampal neurons, and reduces neuronal loss in the APPSwDI/NOS2^−^/^−^ mouse model [40]. It also exhibits anti-prion activity [25], and has been shown to reduce the effects of spinal cord injury, ischemia, and stroke (though not contusive injury), largely attributed to P2 × 7 receptor antagonism and sodium channel modulation [26,41], alongside its neuroprotective and anti-glycemic effects [42,43,44,45]. In oncology, BBG demonstrates both anticancer [46,47] and anti-glioma [48] activity, and it can enhance the fluorescence of other anti-glioma agents, like isorhamnetin [49].

Compared to other glioma markers, such as the glial-transporter–specific dye ASP, which also labels glial cells [31]—BBG displays remarkable specificity, concentrating at or within glioma cells (Figure 3 and Figure 4). Unfortunately, the fluorescence of BBG is not well studied at all and needs many additional experiments to be determined scientifically, including quantum yields and stability [50], and thus it is not easy to compare quantitatively to 5-ALA or SF.

BAP-1 likewise demonstrated exceptional glioma selectivity: thirty minutes post-injection, tumor fluorescence exceeded background by more than 2400-fold in PFA-fixed 15 µm slices and ~85-fold in live 300 µm slices (Figure 5 and Figure 6). Comparing our fluorescence contrast ratios with published results for 5-ALA, our BAP-1 dye showed a glioma-to-background contrast ratio of ~2400 in fine and ~85 in thick slices, which exceeds typical values reported for 5-ALA (~3–20 fold depending on glioma type) [12]. A dose of 0.04 mg provided optimal contrast between tumor tissue and healthy brain parenchyma (Supplementary Figure S2), and the signal decayed almost completely within 24 h, indicating rapid clearance. Residual dye at the intraperitoneal injection site (Supplementary Figure S3E) could complicate subsequent neutron irradiation—issues that may be mitigated by localized cerebral injection or head-only irradiation. Tail-vein administration was ineffective, likely due to first-pass hepatic metabolism [29]. We summarized this data in Supplement Table S1.

In dilute solutions, fluorescence intensity is generally proportional to fluorophore concentration; therefore, the ratio of fluorescence between glioma and surrounding healthy tissue reflects the relative concentration of fluorophore molecules in these regions. However, this ratio may underestimate the true contrast by up to tenfold, as BAP-1 is known to enhance its fluorescence approximately tenfold upon binding to Aβ aggregates [28]. Ultimately, the actual boron concentrations and whether they are sufficient for BNCT can only be determined experimentally under neutron irradiation.

Importantly, BAP-1 is characterized by high photostability and brightness, with a fluorescence half-life spanning several hours, making it suitable for extended imaging sessions [51]. Also, while sodium fluorescein exhibits robust fluorescence and is a widely used fluorescent dye, especially in medical imaging, BODIPY-based dyes like BAP-1 typically have greater fluorescence brightness and photostability on a per-molecule basis, making their fluorescence generally stronger than fluorescein under comparable conditions. The exact fluorescence intensity depends on the experimental setup, dye concentration, and environment. Because each BAP-1 molecule contains only one boron atom, and only about 20% of boron atoms are the B10 isotope, predicting the B10 concentration at the cellular level remains challenging. Direct measurements are necessary to assess the boron load per glioma cell and to determine the biological efficacy of BAP-1 in BNCT, which also depends on the neutron flux (neutrons per second per square meter). Boron biodistribution and boron-10 quantification are planned in follow-up studies using neutron activation analysis and ICP-MS.

Although no acute toxicity or behavioral changes were observed in our short-term mouse studies, comprehensive safety profiling of BAP-1 is essential before considering clinical translation. There may be limitations, including potential toxicity, limited systemic delivery via the tail vein (due to hepatic clearance), and challenges in translation to clinical applications. Anyway, we show that BAP-1 is rapidly cleared from brain tissue within 24 h post-injection, as shown in the Results and Supplementary Figure S4. BBG also demonstrates rapid uptake and clearance. Detailed pharmacokinetic profiling is ongoing and will be reported separately.

## 4. Materials and Methods

### 4.1. Ethics Statement

All procedures involving rodents adhered to the National Institutes of Health guidelines for the ethical use and care of experimental animals and were approved by the Institutional Animal Care and Use Committee (IACUC) of Universidad Central del Caribe (UCC) on 23 March 2023 (protocol #036-2023-06-01). Every effort was made to minimize animal suffering. During surgical procedures, rodents were anesthetized using a Matrix Quanti-Flex VMC anesthesia machine with 4% isoflurane for induction and 1.75% for maintenance. Following the experiments, animals were humanely euthanized for brain tissue collection.

### 4.2. Experimental Animals and Study Design

The purpose of this study was to evaluate tumor fluorescence and glioma-to-background contrast following systemic administration of beta-amyloid-specific dyes: Brilliant Blue G (BBG) and BODIPY-based Amyloid Probe-1 (BAP-1).

Primary outcome: Staining specificity and contrast between glioma tissue and surrounding normal brain tissue. The normal tissue on the same brain slice, imaged under identical excitation parameters, was an internal control; thus, no separate control group was required.

Animals: C57BL/6 mice (Jackson lab, Bar Harbor, ME, USA), 12–16 weeks old, were used for all experiments. Mice were housed under standard laboratory conditions (12-h light/dark cycle, with food and water provided ad libitum). Only animals that developed glioma following intracranial implantation of glioma cells were included in the study.

Experimental groups:

- BBG-treated group: 5 animals received intraperitoneal (IP) BBG injection. A total of 2 animals were used to prepare brain slices containing tumors for post-staining.
- BAP-1-treated groups:

1. 5 animals received a 0.2 mg dose of BAP-1 via IP injection, analyzed 30 min post-injection (GL-261).
2. 5 animals received a 0.04 mg dose of BAP-1 via IP injection, analyzed 30 min post-injection (GL-261).
3. 5 animals received a 0.02 mg dose of BAP-1 via IP injection, analyzed 30 min post-injection (GL-261).
4. 3 animals received a 0.2 mg dose of BAP-1 via IP injection, analyzed 24 h post-injection (GL-261).
5. 3 animals received a 0.2 mg dose of BAP-1 via tail vein injection, analyzed 30 min post-injection (GL-261).
6. 3 animals received a 0.02 mg dose of BAP-1 via IP injection, analyzed 30 min post-injection (KR-158).

A total of 31 animals were used. Sample size was selected based on precedent from similar exploratory studies; no formal power calculation was conducted. Each mouse with a developed glioma served as an individual experimental unit.

Randomization and blinding were not applied due to the exploratory nature of the study. Data analysis was performed by researchers aware of group allocations. Only descriptive statistics (fluorescence ratios and standard error of the mean) were used, in keeping with the study’s exploratory design.

### 4.3. Intracranial Implantation of Glioma Cells

The glioma cells were implanted into the right cerebral hemisphere of 12–16-week-old C57BL/6 mice (Jackson lab), as previously described [31]. After a midline scalp incision was made, a small burr hole (0.5 mm diameter) was drilled into the skull using stereotaxic coordinates (2 mm lateral, 1 mm caudal, and 3 mm ventral to the bregma). A volume of 1 μL of cell suspension (2 × 10^4^ cells/μL in phosphate-buffer solution) was then injected to a depth of 3 mm over 2 min. Sixteen days post-injection, animals were used for the experiments.

### 4.4. Tumor Staining After IPO Injections

Mice were injected IP with either 0.1 mL of 10 mg/mL Brilliant Blue G (BBG, in PBS) (Sigma-Aldrich, St. Louis and Burlington, MA, USA) or 0.1 mL of 2 mg/mL BAP-1 dissolved in 2% polyethylene glycol (PEG-300, Sigma-Aldrich #8.07484) in PBS. BBG and BAP-1 solutions were prepared immediately before the experiment. Thirty minutes after IP injection, animals were euthanized for brain tissue collection (without perfusion).

For BBG staining analysis, brains were removed, fixed in 4% paraformaldehyde, dehydrated through sucrose solution gradients, and cryosectioned at 70 µm using Cryo-M-Bed embedding compound (#720-0094, VWR International, LLC, Manati, PR, USA) and a Vibratome UltraPro 5000 cryostat (Bright Instruments Co., Huntingdon, UK). Tissue sections were mounted with Fluoroshield mounting medium containing 4′,6-diamidino-2-phenylindole (DAPI) (Saint Louis, MO 63103, USA) and examined using confocal and fluorescence microscopy.

For BAP-1 staining analysis, brains were collected intact and sectioned in regions showing visible tumor growth using a Vibrating Blade Microtome (Leica VT100S, Leica, Germany) to produce live brain slices that were 300 µm thick. Slices were mounted in PBS and immediately examined using a confocal microscope.

### 4.5. Glioma Cell Culture

The GL261 and KR158 glioma cell lines, derived from C57BL/6 mice, were obtained from the National Cancer Institute (NCI), Frederick, MD, USA, and the Massachusetts Institute of Technology, Cambridge, MA, USA, respectively. Cells were cultured in Dulbecco’s Modified Eagle’s Medium (DMEM) supplemented with 10% fetal calf serum, 0.2 mM glutamine, and antibiotics (50 U/mL penicillin and 50 μg/mL streptomycin), and maintained in a humidified atmosphere of 5% CO_2_ and 95% air at 37 °C. The culture medium was refreshed every 2–3 days.

### 4.6. Hematoxylin and Eosin Staining

As a control measure, some slices were poststained with hematoxylin-eosin dyes (H&E) (Sigma-Aldrich, Sigma-Aldrich, St. Louis, and Burlington, MA) for additional histological identification of tumors. Briefly, slices were stained in Mayer’s Hematoxylin solution for 1 min, then rinsed with water and then 95% alcohol, counterstained with Eosin Y Solution (alcohol-based) for 1 min, and then dehydrated 2 times with 95% alcohol and 100% alcohol.

### 4.7. Tissue Preparation and Immunofluorescence Staining

Brains were removed, fixed in 4% paraformaldehyde, and cryoprotected by dehydration through graded sucrose solutions. Tissue was embedded in Cryo-M-Bed embedding compound (#720-0094, VWR International, LLC, Puerto Rico) and sectioned at 30 µm using a Vibratome UltraPro 5000 cryostat (Bright Instruments Co., UK). Sections were mounted on glass slides and processed as follows:

Direct BBG staining: Tissue slices were incubated with 0.1 mM BBG in PBS for 30 min, washed briefly in ethanol, and coverslipped with Fluoroshield mounting medium (Sigma-Aldrich, St. Louis, MO, USA; cat. #F6182, DAPI-free).

Immunofluorescence and BBG co-staining: For combined staining, slices were first blocked and permeabilized for 60 min in 5% normal goat serum and 5% normal horse serum (Vector Laboratories, Burlingame, CA, USA) in 0.10 M PBS (NaCl 137 mM; KCl 2.70 mM; Na_2_HPO_4_ 10.14 mM; KH_2_PO_4_ 1.77 mM) containing 0.3% Triton X-100 and 0.05% phenylhydrazine. They were then incubated overnight at 4 °C with a rabbit monoclonal anti-GFAP antibody directly conjugated to Alexa Fluor^®^ 594 (visualized in the Texas Red channel; 1:200; Abcam, Cambridge, MA, USA; cat. no. ab201732), protected from light. Following antibody incubation, slices were washed three times with PBS (10 min each), incubated with 0.1 mM BBG in PBS for 30 min, washed briefly in ethanol, and mounted with Fluoroshield (cat. #F6182, DAPI-free).

### 4.8. Microscopy

Images were acquired either using an Olympus Fluoview FV1000 laser scanning inverted confocal microscope system (Olympus, Melville, NY, USA) or using a simple Olympus CKX53 Inverted Fluorescent Microscope (Olympus, Melville, NY, USA). The images were analyzed using ImageJ software (https://imagej.net/ij, accessed on 24 October 2025) with the Open Microscopy Environment Bio-Formats library and plugin, which enabled the opening of Olympus files (https://docs.openmicroscopy.org/bio-formats/5.4.1/, accessed 24 October 2025). Slides were observed at 10× and 40× magnification using TRITC and Alexa Fluor 687 fluorescence wavelengths. The data were evaluated using custom colorization.

To determine the contrast ratio between glioma areas and healthy tissue, the internal tools of the imaging software Analyze (ImageJ 1.51K, NIH, Bethesda, MD, USA) were used to measure fluorescence intensity within the images. The color values of 10 random points within the glioma region and 10 points within healthy brain tissue were measured. The means of these values were calculated, and the mean glioma-to-healthy tissue ratio and standard error (STR) were determined.

### 4.9. Reagents

BAP-1 was synthesized using the method described previously [27]. BBG was purchased from Sigma-Aldrich (#27815), St. Louis, MO, USA.

### 4.10. Data Analysis

Only descriptive statistics (e.g., ratio calculations, standard error) were applied, consistent with the exploratory objectives of the study.

## 5. Conclusions

The study provides supporting evidence for the presence of Aβ in GBM and highlights the potential of BBG and BAP-1 as candidate fluorescent probes for glioma detection. Following intraperitoneal injection in mice, both compounds demonstrated high tumor specificity and contrast, suggesting their possible utility in future imaging applications. BAP-1′s extended fluorescence half-life may be advantageous for prolonged imaging procedures, while BBG’s potential visibility without microscopy indicates promise for simplifying intraoperative guidance. Further studies are needed to evaluate their safety, biodistribution, and efficacy in clinical settings.

## Data Availability

The original contributions presented in this study are included in the article/Supplementary Material. Further inquiries can be directed to the corresponding author.

## Supplementary Materials

The following supporting information can be downloaded at: https://www.mdpi.com/article/10.3390/ijms262110450/s1, Figures S1–S3. Also, Supplement Table S1, Supplement Validation of Chemical Synthesis, and Supplement with high-resolution originals of Figure 3A–D, and a Supplement of original images for a split-channel confocal montage (Figure 2). Figure S1: BBG dye accumulates in brain tumors of the C57Bl/6-GL261 mouse glioma model, resulting in visible brown staining of the tumor tissue. The image shows a 70 µm frozen brain section containing an implanted tumor, mounted on a cryostat microtome plate at −23 °C. The brain was analyzed 30 min after intraperitoneal injection of 100 µL of BBG (10 mg/mL in PBS). Scale bar: 1 mm. Figure S2: Confocal imaging of BAP-1–stained glioma in live mouse brain sections using 0.04 mg and 0.02 mg doses of BAP-1. Selective staining of glioma tumors in the C57BL/6-GL261 mouse model was visualized as green fluorescence 30 min after intraperitoneal injection of BAP-1. Live brain sections (300 µm thick) were prepared 30 min after IP injection of 100 µL BAP-1 (0.4 mg/mL or 0.2 mg/mL in 2% polyethylene glycol [PEG-300] in PBS). Images were acquired using TRITC HYQ filters (excitation: 530–560 nm; emission: 590–650 nm). Tumor cells appear green. Scale bar: 10 µm. Figure S3: BAP-1 distribution following intraperitoneal and tail-vein injection: residual accumulation at the injection site and reduced glioma staining after systemic administration. Peritoneum staining at the site of injection (A) and glioma tumor staining (B) in the C57BL/6-GL261 mouse model were visualized as green fluorescence 30 min after BAP-1 injection. Live tissue sections (300 µm thick) were prepared 30 min after IP injection of 100 µL BAP-1 (2 mg/mL in 2% polyethylene glycol [PEG-300] in PBS). Images were acquired using TRITC HYQ filters (excitation: 530–560 nm; emission: 590–650 nm). Tumor cells appear green. Scale bar: 10 µm. Table S1: Staining specificity and contrast between glioma tissue and surrounding normal brain tissue.

## Abbreviations

The following abbreviations are used in this manuscript:

5-ALA	5-aminolevulenic acid
APP	amyloid precursor protein
Aβ	amyloid beta
BAP-1	BODIPY-based Amyloid Probe-1
BBG	Brilliant Blue G
BBB	Blood-brain barrier
BNCT	boron-neutron capture therapy
GBM	glioblastoma
GTR	Gross total resection
H&E	hematoxylin and eosin
HGG	high-grade gliomas
ICG	indocyanine green
ICP-MS	Inductively coupled plasma Mass-Spec
IP	intraperitoneal
PpIX	protoporphyrin IX
SF	fluorescein sodium
