# Supplementary material for: Targeting Gliomas with Beta-Amyloid-Specific Dyes: A Novel Approach for In Vivo Staining and Potential Therapeutic Applications
Source: Int J Mol Sci. 2025 Oct 28;26(21):10450. doi: 10.3390/ijms262110450 (PMC12607655; doi:10.3390/ijms262110450)
Supplement: Supplementary file 1 [file ijms-26-10450-s001.zip › Table S1.pdf]

Supplement Table S1. Fluorescence and standard error of the mean in 300 micron live brain slices in mice after BAP-1 injection, and ratios. For representative images of these experiments see: Figure 4, Figures S2 and S3

Ratio	Tumor core	Healthy brain	Time after IP	BAP-1 dose	Cell type	Animals/group
10.39:1	119.5±20.3	11.5±3.0	30 min	0.2 mg	GL-261	N=5
29.95	59.9±20.9	2.0±0.76	30 min	0.02 mg	GL-261	N=5
85.85- Best	111.6±16.7	1.3±0.9	30 min	0.04 mg	GL-261	N=5
24.36	87.2±13.5	3.58±0.91	30 min	0.02 mg	KR-158	N=3
2.46:1	12.31±1.5	5±0.3	24 h	0.2 mg	GL-261	N=3
			Time after tail injection			
N/A	27.17±15.4	n	30 min	0.2 mg	GL-261	N=3
