# Supplementary material for: Targeting Gliomas with Beta-Amyloid-Specific Dyes: A Novel Approach for In Vivo Staining and Potential Therapeutic Applications
Source: Int J Mol Sci. 2025 Oct 28;26(21):10450. doi: 10.3390/ijms262110450 (PMC12607655; doi:10.3390/ijms262110450)
Supplement: Supplementary file 1 [file ijms-26-10450-s001.zip › Validadtion of the sinthesis BAP-1/BAP-1 13C.pdf]

Boris ERNOLINSKY

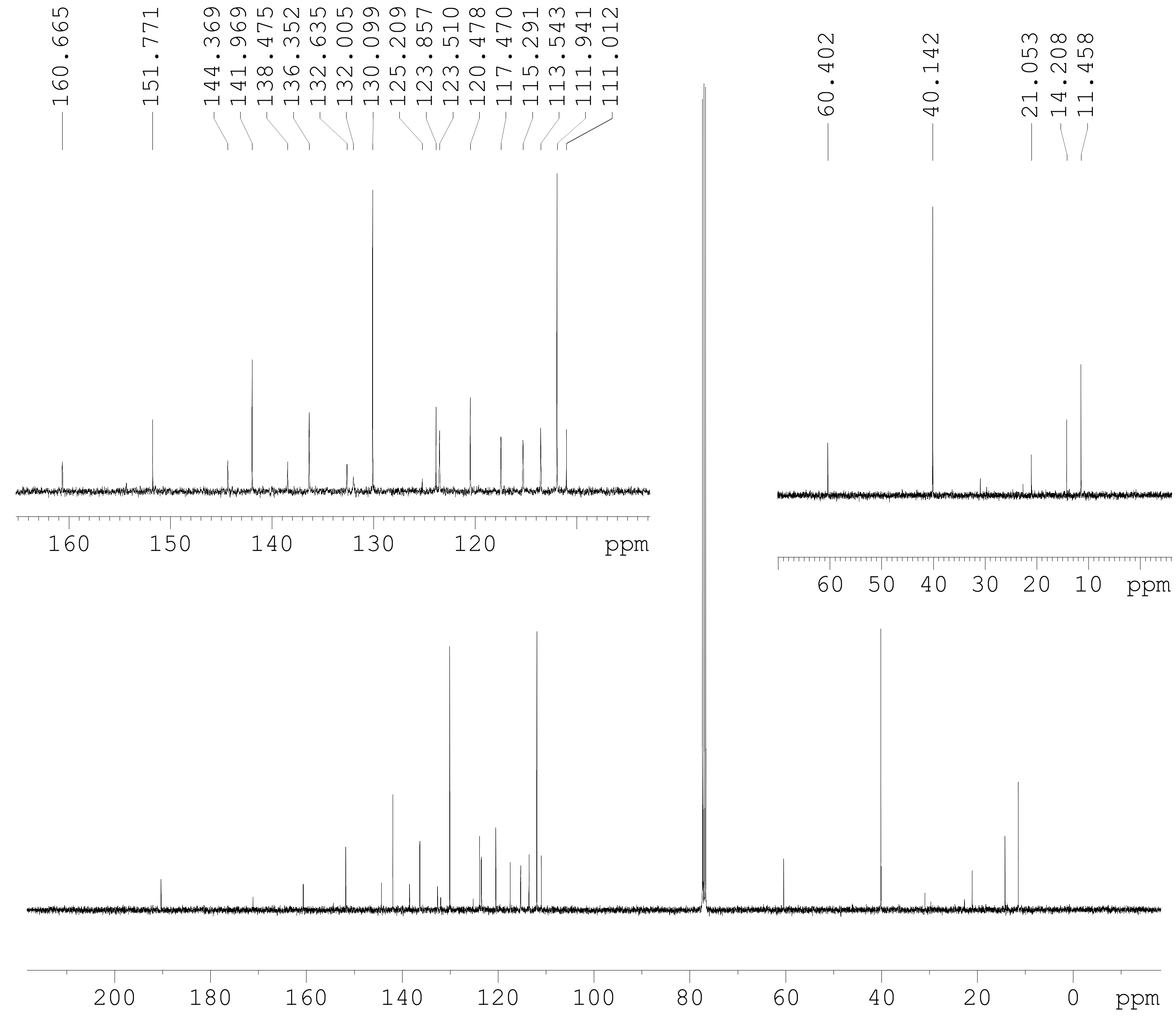

Current Data Parameters

NAME	BAP1	
EXPNO	7	
PROCNO	1	

F2 - Acquisition Parameters

Date_	20230110	
Time	13.16	h
INSTRUM	Avance Core	
PROBHD	Z178277_0019 (	
PULPROG	zgpg30	
TD	47618	
SOLVENT	CDCl3	
NS	1024	
DS	0	
SWH	23809.523	Hz
FIDRES	1.000022	Hz
AQ	0.9999780	sec
RG	101	
DW	21.000	usec
DE	6.50	usec
TE	299.7	K
D1	1.00000000	sec
D11	0.03000000	sec
TD0	1	
SFO1	100.6228298	MHz
NUC1	13C	
P0	3.33	usec
P1	10.00	usec
PLW1	59.77799988	W
SFO2	400.1316005	MHz
NUC2	1H	
CPDPRG[2	waltz65	
PCPD2	90.00	usec
PLW2	13.75000000	W
PLW12	0.16975001	W
PLW13	0.08538500	W

F2 - Processing parameters

SI	32768	
SF	100.6127685	MHz
WDW	EM	
SSB	0	
LB	1.00	Hz
GB	0	
PC	1.40	
