# Supplementary material for: Targeting Gliomas with Beta-Amyloid-Specific Dyes: A Novel Approach for In Vivo Staining and Potential Therapeutic Applications
Source: Int J Mol Sci. 2025 Oct 28;26(21):10450. doi: 10.3390/ijms262110450 (PMC12607655; doi:10.3390/ijms262110450)
Supplement: Supplementary file 1 [file ijms-26-10450-s001.zip › Validadtion of the sinthesis BAP-1/precursor-1H.pdf]

Boris ERNOLINSKY

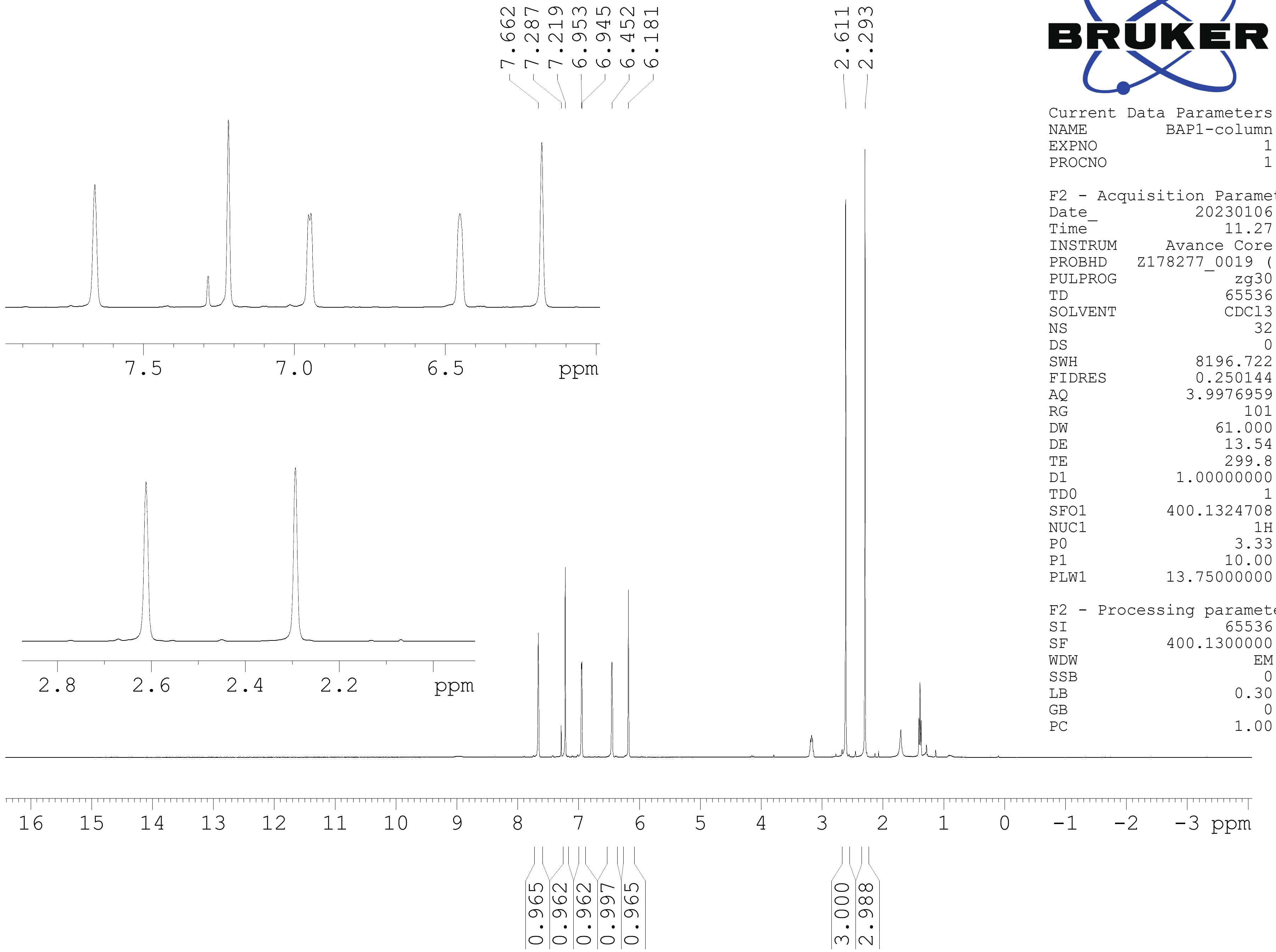

Current Data Parameters

NAME	BAP1-column
EXPNO	1
PROCNO	1

F2 - Acquisition Parameters

Date_	20230106
Time	11.27 h
INSTRUM	Avance Core
PROBHD	Z178277_0019 (
PULPROG	zg30
TD	65536
SOLVENT	CDCl3
NS	32
DS	0
SWH	8196.722 Hz
FIDRES	0.250144 Hz
AQ	3.9976959 sec
RG	101
DW	61.000 usec
DE	13.54 usec
TE	299.8 K
D1	1.00000000 sec
TD0	1
SFO1	400.1324708 MHz
NUC1	1H
P0	3.33 usec
P1	10.00 usec
PLW1	13.75000000 W

F2 - Processing parameters

SI	65536
SF	400.1300000 MHz
WDW	EM
SSB	0
LB	0.30 Hz
GB	0
PC	1.00
