# Supplementary figures and images for: Targeting Gliomas with Beta-Amyloid-Specific Dyes: A Novel Approach for In Vivo Staining and Potential Therapeutic Applications
Source: Int J Mol Sci. 2025 Oct 28;26(21):10450. doi: 10.3390/ijms262110450 (PMC12607655; doi:10.3390/ijms262110450)

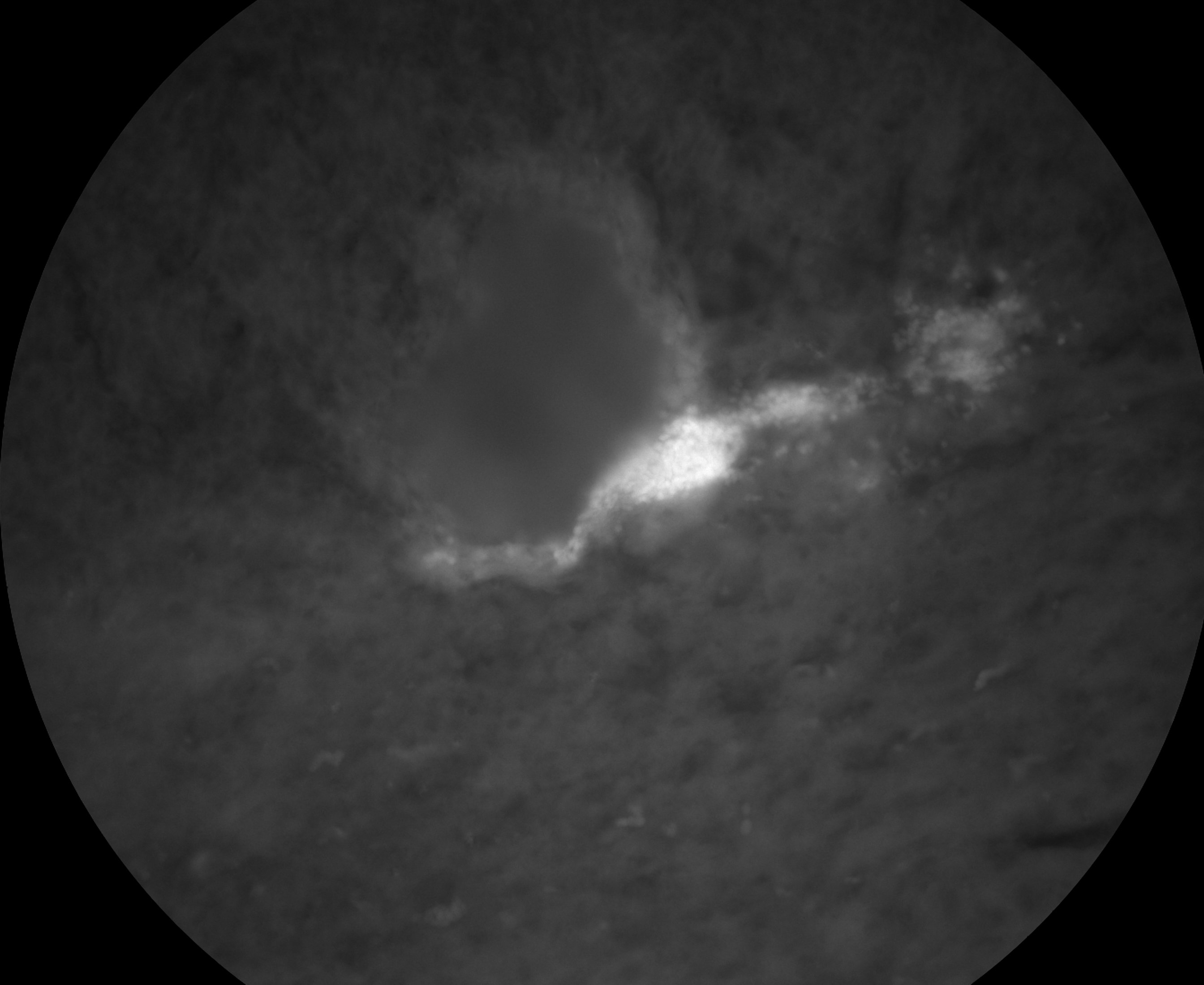

Supplement: Supplementary file 1 [file ijms-26-10450-s001.zip › Originals Fig3 A,B,C,D/Fig 3A red chann B-W.jpg]

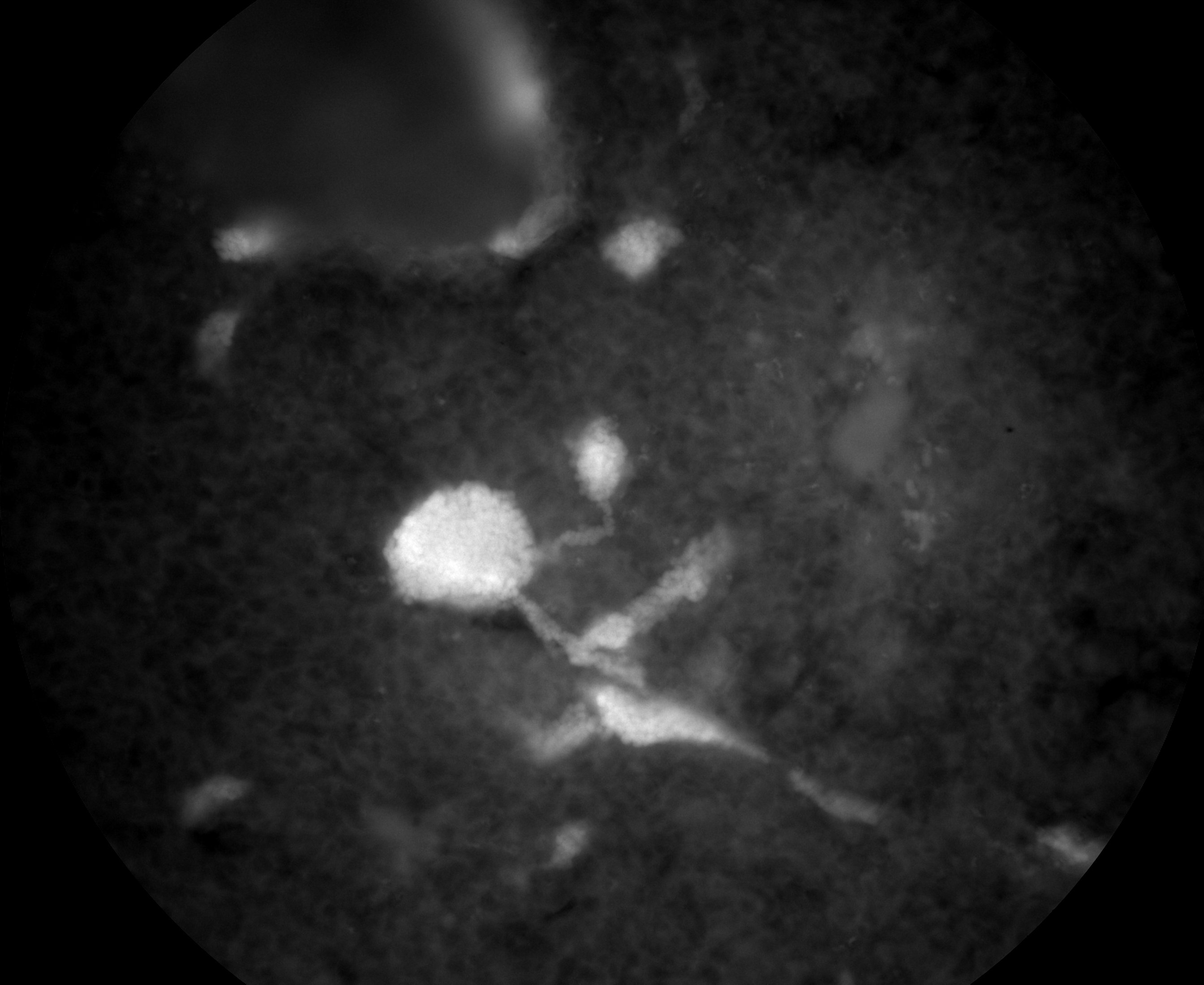

Supplement: Supplementary file 1 [file ijms-26-10450-s001.zip › Originals Fig3 A,B,C,D/Fig 3B red chann B-W.jpg]

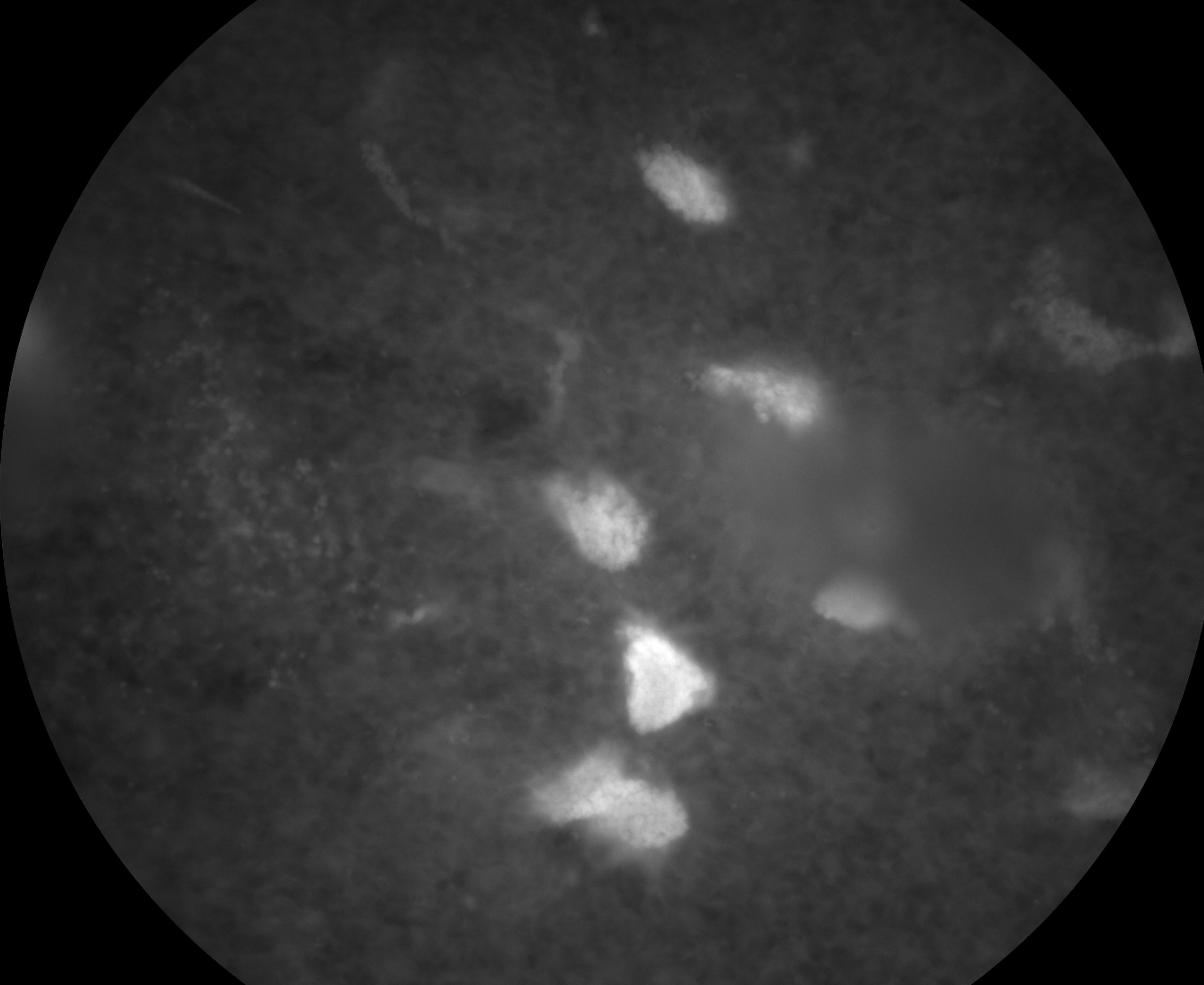

Supplement: Supplementary file 1 [file ijms-26-10450-s001.zip › Originals Fig3 A,B,C,D/Fig 3C red chann B-W.jpg]

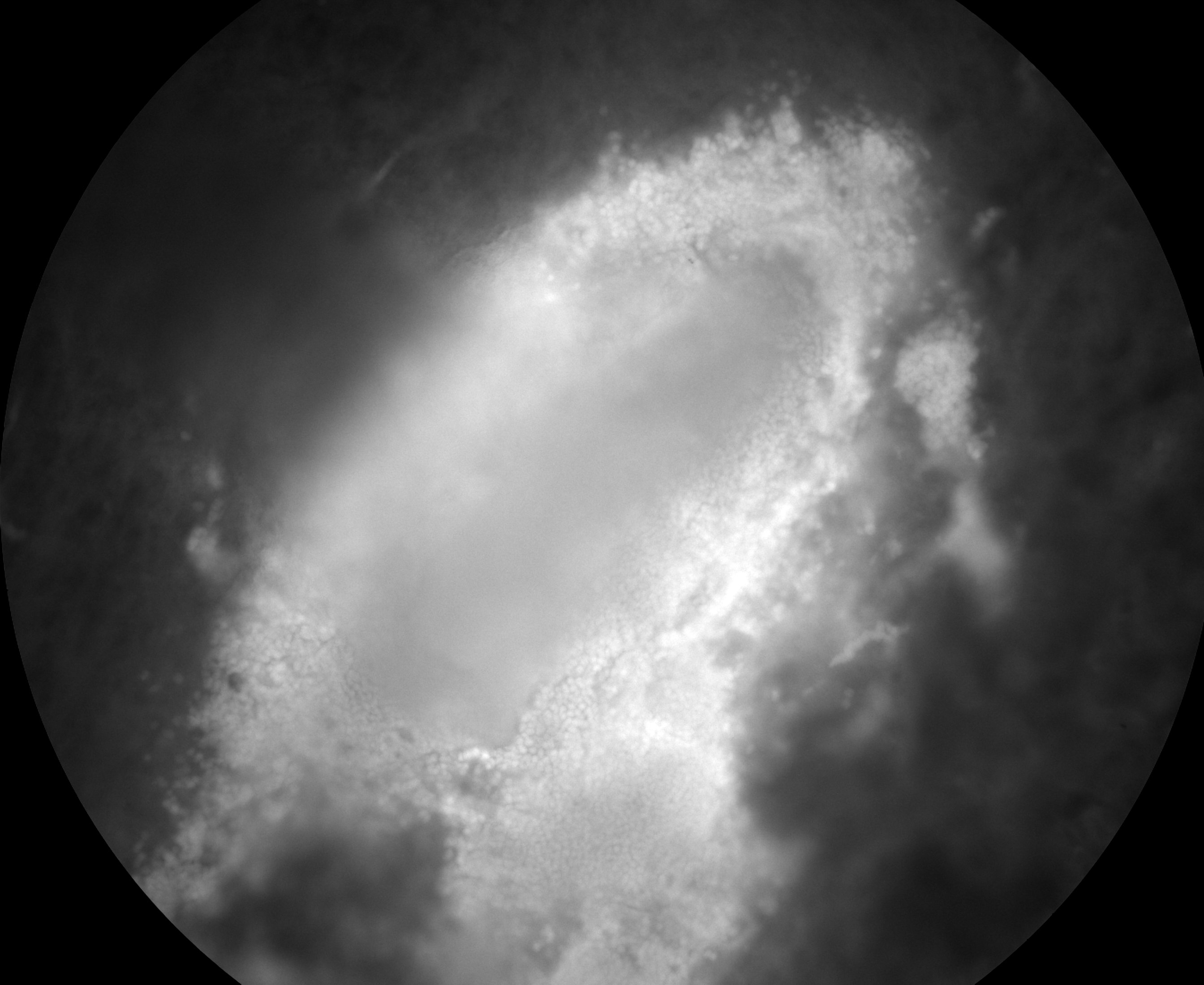

Supplement: Supplementary file 1 [file ijms-26-10450-s001.zip › Originals Fig3 A,B,C,D/Fig 3D red chann B-W.jpg]

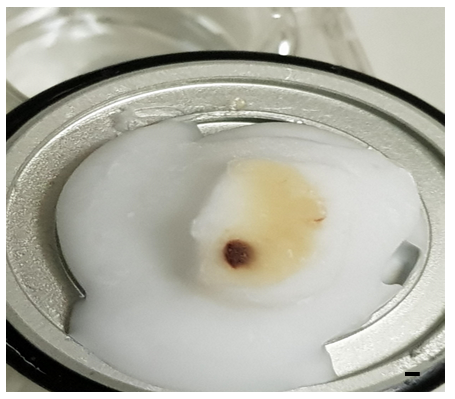

Supplement: Supplementary file 1 [file ijms-26-10450-s001.zip › S1.tif]

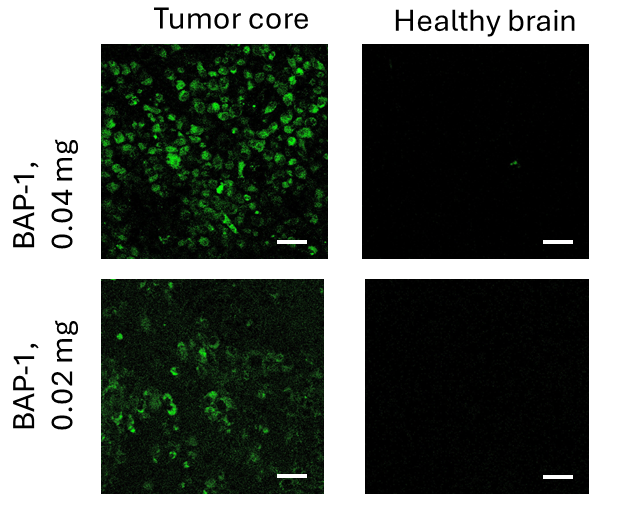

Supplement: Supplementary file 1 [file ijms-26-10450-s001.zip › S2.tif]

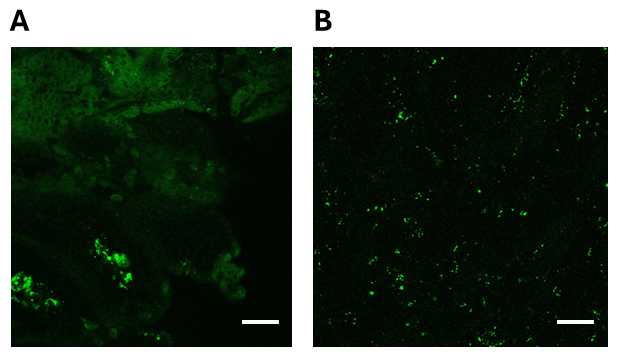

Supplement: Supplementary file 1 [file ijms-26-10450-s001.zip › S3.tif]

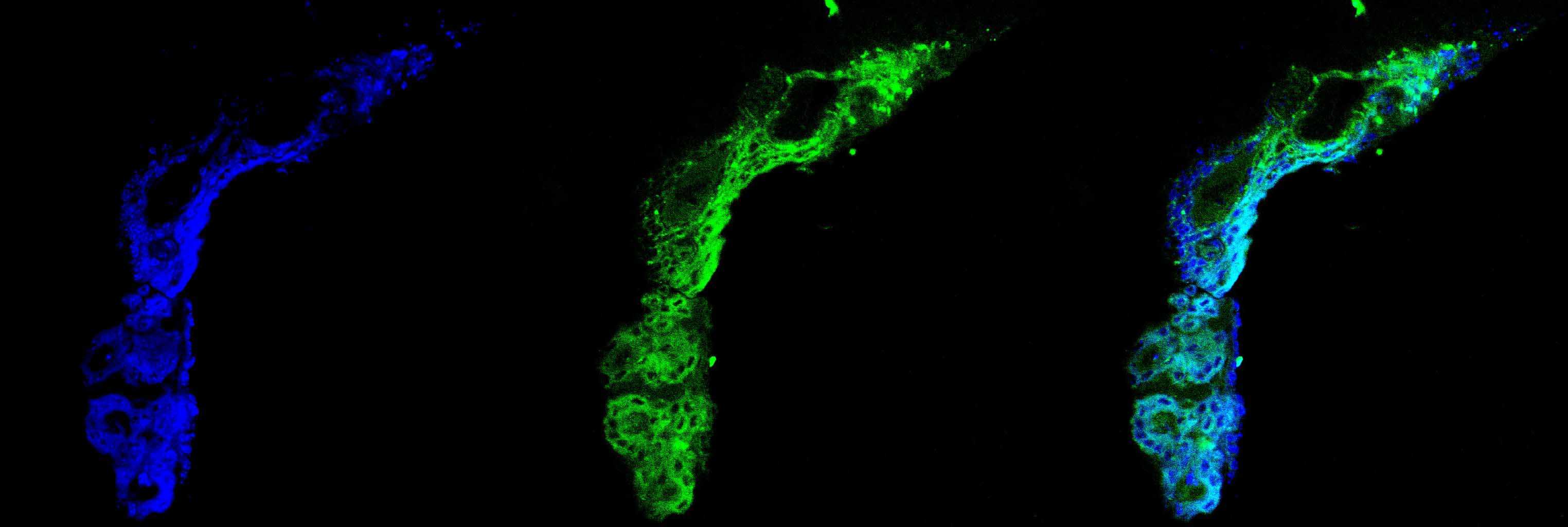

Supplement: Supplementary file 1 [file ijms-26-10450-s001.zip › Split DAPI and BAP-1 composite/Image0009 x40 625-650 nm composite DAPI+BAP-1.jpg]

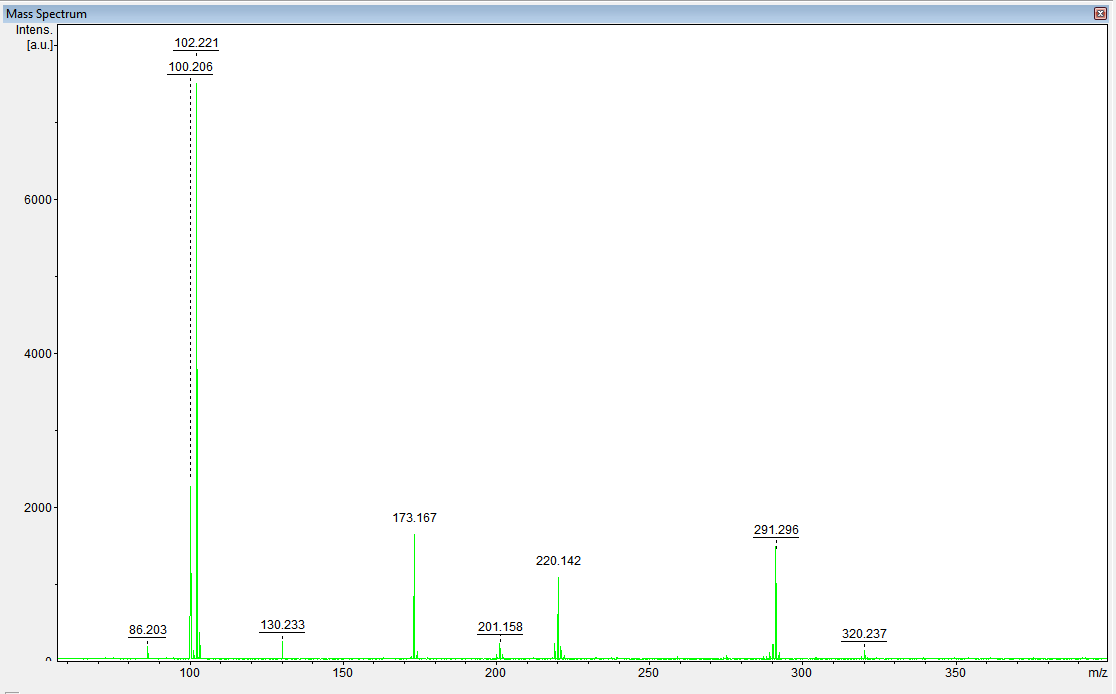

Supplement: Supplementary file 1 [file ijms-26-10450-s001.zip › Validadtion of the sinthesis BAP-1/mass 220.PNG]
